# Ideals and their complements in commutative semirings

**DOI:** 10.1007/s00500-018-3493-2

**Published:** 2018-08-31

**Authors:** Ivan Chajda, Helmut Länger

**Affiliations:** 1 Department of Algebra and Geometry, Faculty of Science, Palacký University Olomouc, 17. listopadu 12, 771 46 Olomouc, Czech Republic; 2 Faculty of Mathematics and Geoinformation, Institute of Discrete Mathematics and Geometry, TU Wien, Wiedner Hauptstraße 8-10, 1040 Vienna, Austria

**Keywords:** Commutative semiring, Idempotent semiring, Ideal, Annihilator, Lattice of ideals, Complement, Łukasiewicz semiring

## Abstract

We study conditions under which the lattice ${{\mathrm{\mathbf {Id}}}}\mathbf R$ of ideals of a given a commutative semiring ${\mathbf {R}}$ is complemented. At first we check when the annihilator $I^*$ of a given ideal *I* of ${\mathbf {R}}$ is a complement of *I*. Further, we study complements of annihilator ideals. Next we investigate so-called Łukasiewicz semirings. These form a counterpart to MV-algebras which are used in quantum structures as they form an algebraic semantic of many-valued logics as well as of the logic of quantum mechanics. We describe ideals and congruence kernels of these semirings with involution. Finally, using finite unitary Boolean rings, a construction of commutative semirings with complemented lattice of ideals is presented.

## Introduction

Semirings play an important role in both algebra and applications. They share several important properties of rings, but, on the other hand, every distributive lattice with the least element can be recognized as an idempotent semiring. Hence, if the addition operation of a semiring is idempotent, then the semiring often shares some properties with semilattices or lattices. Contrary to rings, ideals of semirings need not be zero-classes of congruences, so-called congruence kernels. However, the set of all ideals forms a complete lattice similarly as for rings. We study under what conditions this lattice is complemented. It turns out that for commutative semirings having no 2-nilpotent element, one complement of a given ideal is given by its annihilator. Analogous results are obtained for the lattice of annihilator ideals though this lattice need not be a sublattice of the lattice of ideals.

In the second part we study certain semirings with involution, so-called Łukasiewicz semirings and their ideals. These semirings originated in the study of quantum structures, see, for example, Bonzio et al. (2016) and Chajda et al. (2018) for the concepts and motivation. The ideals of Łukasiewicz semirings have interesting properties, and the congruence kernels of these semirings can be described easily. Complements within the lattice of ideals can be described by means of complemented elements.

It might be the case that commutative semirings are complemented but their complements do not coincide with the corresponding annihilators. We show that by forming the direct product of such semiring with a finite unitary Boolean ring we obtain a commutative semiring whose lattice of ideals has similar properties as the lattice of ideals of the original semiring. Here we use the fact that in such a case we have no skew ideals in the direct product and hence the lattice of ideals of the new semiring is the direct product of the lattices of ideals of its factors.

## Basic concepts

In the literature there exist different definitions of a semiring. We will use the following one which differs from that in Golan (1999) (where it is called a “hemiring”).

A *commutative semiring* is an algebra ${\mathbf {R}}=(R,+,\cdot ,0)$ of type (2, 2, 0) such that

- $(R,+,0)$ is a commutative monoid,
- $(R,\cdot )$ is a commutative semigroup,
- $$(x+y)\cdot z\approx x\cdot z+y\cdot z$$,
- $$0\cdot x\approx 0$$.

If there exists an element 1 of *R* satisfying the identity $x\cdot 1\approx x$ then ${\mathbf {R}}$ is called *unitary*, and if ${\mathbf {R}}$ satisfies the identity $x+x\approx x$ then ${\mathbf {R}}$ is called *idempotent*.

For subsets *A*, *B* of *R* put

$$\begin{aligned} A+B&:=\{x+y\mid x\in A,y\in B\}, \\ AB&:=\{x\cdot y\mid x\in A,y\in B\}. \end{aligned}$$

For $a\in R$ we write *aA* instead of $\{a\}A$ etc.

An *ideal* of ${\mathbf {R}}$ is a subset *I* of *R* satisfying $0\in I$, $I+I\subseteq I$ and $IR\subseteq I$. Let ${{\mathrm{Id}}}{\mathbf {R}}$ denote the set of all ideals of ${\mathbf {R}}$. Since ${{\mathrm{Id}}}{\mathbf {R}}$ is closed with respect to arbitrary set-theoretical intersections, ${{\mathrm{\mathbf {Id}}}}{\mathbf {R}}:=({{\mathrm{Id}}}R,\subseteq )$ is a complete lattice with the least element $\{0\}$ and the greatest element *R*. Obviously, the lattice operations are given by $+$ and $\cap$. More generally, in ${{\mathrm{\mathbf {Id}}}}{\mathbf {R}}$ we have

$$\begin{aligned} \bigvee _{s\in S}I_s&=\sum _{s\in S}I_s, \\ \bigwedge _{s\in S}I_s&=\bigcap _{s\in S}I_s \end{aligned}$$

where $\sum \nolimits _{s\in S}I_s$ denotes the set of all sums of finitely many elements of $\bigcup \nolimits _{s\in S}I_s$.

In the following we will write *xy* instead of $x\cdot y$ and we will denote the set of all nonnegative integers by ${\mathbb {N}}_0$.

### Example 2.1

Every commutative ring is a commutative semiring. $\mathbf N_0:=({\mathbb {N}}_0,+,\cdot ,0)$ is a commutative semiring which is not a ring since 1 has no additive inverse. Further, $2\mathbb N_0\in {{\mathrm{Id}}}{\mathbf {N}}_0$. Every distributive lattice with 0 is a commutative semiring which is a ring only if it is a singleton. If ${\mathbf {L}}_1=(L_1,\vee ,\wedge ,0)$ and $\mathbf L_2=(L_2,\vee ,\wedge ,0)$ are distributive lattices with 0 then $\{0\}\times L_2\in {{\mathrm{Id}}}({\mathbf {L}}_1\times {\mathbf {L}}_2)$.

In order to study the structure of lattices of ideals, we remember several concepts from lattice theory.

Recall that a lattice $(L,\vee ,\wedge )$ is called *modular* if it satisfies the identity

$$\begin{aligned} (x\vee y)\wedge (x\vee z)\approx x\vee (y\wedge (x\vee z)). \end{aligned}$$

It is well known that lattices of ideals of rings are modular. Unfortunately, this need not hold for lattices of ideals of semirings, as the following example shows.

### Example 2.2

**Fig. 1:**
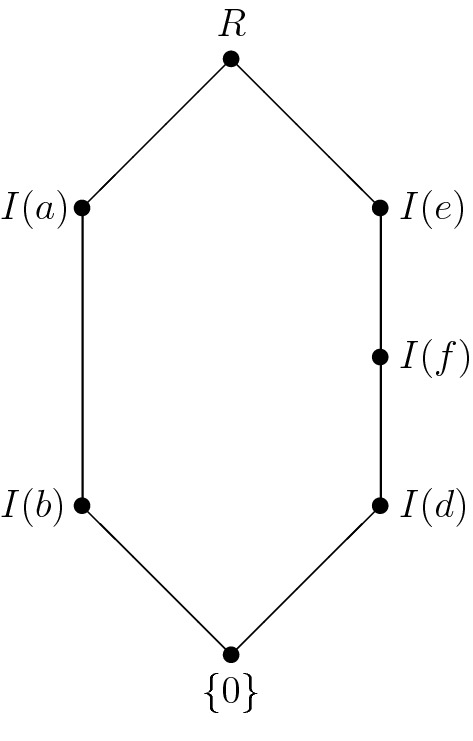
The lattice of ideals of $\mathbf R$ from Example 2.2

Let ${\mathbf {R}}=(R,+,\cdot ,0)$ denote the commutative semiring defined by $R:=\{0,a,b,c,d,e,f,g\}$,

$$\begin{aligned} \begin{array}{c|cccccccc} + &{} \quad 0 &{}\quad a &{}\quad b &{}\quad c &{}\quad d &{}\quad e &{}\quad f &{}\quad g \\ \hline 0 &{}\quad 0 &{}\quad a &{}\quad b &{}\quad c &{}\quad d &{}\quad e &{}\quad f &{}\quad g \\ a &{}\quad a &{}\quad b &{}\quad c &{}\quad 0 &{}\quad e &{}\quad f &{}\quad g &{}\quad d \\ b &{}\quad b &{}\quad c &{}\quad 0 &{}\quad a &{}\quad f &{}\quad g &{}\quad d &{}\quad e \\ c &{}\quad c &{}\quad 0 &{}\quad a &{}\quad b &{}\quad g &{}\quad d &{}\quad e &{}\quad f \\ d &{}\quad d &{}\quad e &{}\quad f &{}\quad g &{}\quad d &{}\quad e &{}\quad f &{}\quad g \\ e &{}\quad e &{}\quad f &{}\quad g &{}\quad d &{}\quad e &{}\quad f &{}\quad g &{}\quad d \\ f &{}\quad f &{}\quad g &{}\quad d &{}\quad e &{}\quad f &{}\quad g &{}\quad d &{}\quad e \\ g &{}\quad g &{}\quad d &{}\quad e &{}\quad f &{}\quad g &{}\quad d &{}\quad e &{}\quad f \end{array} \end{aligned}$$

and $xy:=0$ for all $x,y\in R$. Evidently, ${\mathbf {R}}$ is not a ring. ${\mathbf {R}}$ has the ideals $\{0\},I(a),I(b),$*I*(*d*), *I*(*e*), *I*(*f*), *R* where $I(a):=\{0,a,b,c\}$, $I(b):=\{0,b\}$, $I(d):=\{0,d\}$, $I(e):=\{0,d,e,f,g\}$ and $I(f):=\{0,d,f\}$. The Hasse diagram of ${{\mathrm{\mathbf {Id}}}}{\mathbf {R}}$ is shown in Fig. 1.

Obviously, for an arbitrary subset *A* of *R* the set

$$\begin{aligned} I(A)&:=\{a_1r_1+\cdots +a_kr_k+n_1a_{k+1}+\cdots +n_la_{k+l}\mid k,l\\&\ge 0,a_1,\ldots ,a_{k+l}\in A, r_1,\ldots ,r_k\in R,n_1,\ldots ,n_l\in {\mathbb {N}}_0\} \end{aligned}$$

is the ideal generated by *A*. Here, for arbitrary $a\in R$ we have

$$\begin{aligned} 0a:=0,\ 1a:=a,\ 2a:=a+a,\ldots . \end{aligned}$$

Particularly, for $a\in R$ the set

$$\begin{aligned} I(a):=aR+{\mathbb {N}}_0a \end{aligned}$$

(where ${\mathbb {N}}_0a$ denotes the set $\{na\mid n\in {\mathbb {N}}_0\}$) is the ideal generated by *a*. Such *ideals* are called *principal*.

## Annihilators as complements of ideals

For any $I\in {{\mathrm{Id}}}{\mathbf {R}}$ we define $I^*:=\{x\in R\mid xI=\{0\}\}$, call $I^*$ an *annihilator ideal* of ${\mathbf {R}}$ and $^*$*annihilation* or *annihilation mapping* and put ${{\mathrm{Ann}}}{\mathbf {R}}:=\{I^*\mid I\in {{\mathrm{Id}}}{\mathbf {R}}\}$. It is easy to see that ${{\mathrm{Ann}}}{\mathbf {R}}\subseteq {{\mathrm{Id}}}{\mathbf {R}}$. Moreover, ${{\mathrm{\mathbf {Ann}}}}{\mathbf {R}}:=({{\mathrm{Ann}}}{\mathbf {R}},\subseteq )$ is a complete lattice. This follows from (i) of Lemma 3.3.

### Example 3.1

Referring to Example 2.1 we have $(2{\mathbb {N}}_0)^*=\{0\}$ and $(\{0\}\times L_2)^*=L_1\times \{0\}$ provided $L_2$ is not a singleton.

The following facts are straightforward.

### Lemma 3.2

Let ${\mathbf {R}}=(R,+,\cdot ,0)$ be a commutative semiring and $I,J\in {{\mathrm{Id}}}{\mathbf {R}}$. Then

- $$I\subseteq J$$ implies $$J^*\subseteq I^*$$,
- $$I\subseteq I^{**}$$,
- $$I^{***}=I^*$$,
- $$\{0\}^*=R$$,
- if ${\mathbf {R}}$ is unitary then $R^*=\{0\}$.

From Lemma 3.2 we obtain $${{\mathrm{Ann}}}{\mathbf { R}}=\{I\in {{\mathrm{Id}}}{\mathbf {R}}\mid I^{**}=I\}$$.

In the following we are going to investigate the lattice operations in ${{\mathrm{\mathbf {Id}}}}{\mathbf {R}}$ and ${{\mathrm{\mathbf {Ann}}}}{\mathbf {R}}$. Since these lattices are complete, we consider, more generally, infinite joins and meets.

### Lemma 3.3

Let ${\mathbf {R}}=(R,+,\cdot ,0)$ be a commutative semiring. In ${{\mathrm{\mathbf {Id}}}}{\mathbf {R}}$ we have

(i) $$\left( \sum \nolimits _{s\in S}I_s\right) ^*=\bigcap \nolimits _{s\in S}I_s^*$$,
(ii) $$\left( \bigcap \nolimits _{s\in S}I_s\right) ^*\supseteq \sum \nolimits _{s\in S}I_s^*$$.

### Proof

(i) We have $I_t\subseteq \sum \nolimits _{s\in S}I_s$ for all $t\in S$ and hence $(\sum \nolimits _{s\in S}I_s)^*\subseteq I_t^*$ for all $t\in S$ whence $(\sum \nolimits _{s\in S}I_s)^*\subseteq \bigcap \nolimits _{s\in S}I_s^*$. On the other hand, if $a\in \bigcap \nolimits _{s\in S}I_s^*$ and $b\in \sum \nolimits _{s\in S}I_s$ then there exist $b_1,\ldots ,b_n\in \bigcup \nolimits _{s\in S}I_s$ with $b_1+\cdots +b_n=b$ and hence
  $$\begin{aligned} ab= & {} a(b_1+\cdots +b_n)=ab_1+\cdots +ab_n\\= & {} 0+\cdots +0=0, \end{aligned}$$
  i.e. $a\in (\sum \nolimits _{s\in S}I_s)^*$ showing $\bigcap \nolimits _{s\in S}I_s^*\subseteq (\sum \nolimits _{s\in S}I_s)^*$ and hence (i).
(ii) We have $\bigcap \nolimits _{s\in S}I_s\subseteq I_t$ for all $t\in S$ and hence $I_t^*\subseteq (\bigcap \nolimits _{s\in S}I_s)^*$ for all $t\in S$ which shows (ii).

$$\square $$

As remarked above, since in ${{\mathrm{Ann}}}{\mathbf {R}}$ we have

1 $$\begin{aligned} \bigcap _{s\in S}I_s=\bigcap _{s\in S}I_s^{**}=(\sum _{s\in S}I_s^*)^*\in {{\mathrm{Ann}}}{\mathbf {R}}, \end{aligned}$$

${{\mathrm{\mathbf {Ann}}}}{\mathbf {R}}$ is a complete lattice.

The following lemma shows that in general the supremum within ${{\mathrm{\mathbf {Ann}}}}{\mathbf {R}}$ may differ from that within ${{\mathrm{\mathbf {Id}}}}{\mathbf {R}}$.

### Lemma 3.4

Let ${\mathbf {R}}=(R,+,\cdot ,0)$ be a commutative semiring. In ${{\mathrm{\mathbf {Ann}}}}{\mathbf {R}}$ we have

2 $$\begin{aligned} \bigvee _{s\in S}I_s&=\left( \sum _{s\in S}I_s\right) ^{**}, \end{aligned}$$

3 $$\begin{aligned} \bigwedge _{s\in S}I_s&=\bigcap _{s\in S}I_s. \end{aligned}$$

### Proof

For all $$t\in S$$ we have $$I_t\subseteq \sum \nolimits _{s\in S}I_s$$ and hence $$(\sum \nolimits _{s\in S}I_s)^*\subseteq I_t^*$$ whence $$I_t=I_t^{**}\subseteq (\sum \nolimits _{s\in S}I_s)^{**}$$. Moreover, if $$I\in {{\mathrm{Ann}}}{\mathbf {R}}$$ and $$I_s\subseteq I$$ for all $$s\in S$$ then $$\sum \nolimits _{s\in S}I_s\subseteq I$$ and hence $$I^*\subseteq (\sum \nolimits _{s\in S}I_s)^*$$ whence $$(\sum \nolimits _{s\in S}I_s)^{**}\subseteq I^{**}=I$$. This proves (2). Finally, according to (1), $${{\mathrm{Ann}}}{\mathbf {R}}$$ is closed under arbitrary intersections which yields (3). $$\square $$

The lattice of ideals and the annihilator lattice of ${\mathbf {R}}$ can be written in the form

$$\begin{aligned} {{\mathrm{\mathbf {Id}}}}{\mathbf {R}}&=({{\mathrm{Id}}}{\mathbf {R}},+,\cap ,\{0\},R), \\ {{\mathrm{\mathbf {Ann}}}}{\mathbf {R}}&=({{\mathrm{Ann}}}{\mathbf {R}},\vee ,\cap ,R^*,R), \end{aligned}$$

respectively, where

$$\begin{aligned} I\vee J:=(I+J)^{**} \end{aligned}$$

for all $$I,J\in {{\mathrm{Ann}}}{\mathbf {R}}$$.

If for an arbitrary subset *A* of *R* we define $A^*:=\{x\in R\mid xA=\{0\}\}$, then $A^*=I(A)^*$ and hence ${{\mathrm{Ann}}}{\mathbf {R}}=\{A^*\mid A\subseteq R\}$.

Now, we define the concept which plays a crucial role for complementation in ${{\mathrm{\mathbf {Id}}}}{\mathbf {R}}$.

### Definition 3.5

We call an element *a* of a commutative semiring ${\mathbf {R}}=(R,+,\cdot ,0)$ 2-*nilpotent* if $a\ne 0=a^2$.

Recall that an *ortholattice* (see Birkhoff 1979) is an algebra $(L,\vee ,\wedge ,',0,1)$ of type (2, 2, 1, 0, 0) such that $(L,\vee ,\wedge ,0,1)$ is a bounded lattice and the following identities are satisfied:

$$\begin{aligned} x \vee x'\approx & {} 1 ,\ x\wedge x'\approx 0,\ (x')'\approx x,\ (x\vee y)'\approx x'\wedge y',\ \\&(x\wedge y)'\approx x'\vee y'. \end{aligned}$$

Hence, in every ortholattice we have $x\le y$ if and only if $y'\le x'$. Such a complementation is called an *orthocomplementation*, see Birkhoff (1979).

In the following we set

$$\begin{aligned} {{\mathrm{\mathbf {Id}}}}^*{\mathbf {R}}&:=({{\mathrm{Id}}}{\mathbf {R}},+,\cap ,{}^*,\{0\},R)\text { and} \\ {{\mathrm{\mathbf {Ann}}}}^*{\mathbf {R}}&:=({{\mathrm{Ann}}}{\mathbf {R}},\vee ,\cap ,{}^*,R^*,R) \end{aligned}$$

and investigate under which conditions these algebras are ortholattices. Obviously,

$$\begin{aligned} {{\mathrm{\mathbf {Id}}}}^*{\mathbf {R}}={{\mathrm{\mathbf {Ann}}}}^*{\mathbf {R}}\text { if and only it }I^{**}=I\text { for all }I\in {{\mathrm{Id}}}{\mathbf {R}}. \end{aligned}$$

### Lemma 3.6

Let ${\mathbf {R}}=(R,+,\cdot ,0)$ be a commutative semiring. Then for every ideal *I* of ${\mathbf {R}}$ its pseudocomplement in ${{\mathrm{\mathbf {Id}}}}{\mathbf {R}}$ is just $I^*$ if and only if ${\mathbf {R}}$ has no 2-nilpotent element.

### Proof

First assume $I^*$ to be the pseudocomplement of *I* in ${{\mathrm{\mathbf {Id}}}}{\mathbf {R}}$ for each $I\in {{\mathrm{Id}}}{\mathbf {R}}$. If $a\in R$ and $a^2=0$ then $a\in I(a)\cap I(a)^*=\{0\}$ and hence $a=0$. Conversely, assume ${\mathbf {R}}$ to have no 2-nilpotent element. Let $J,K\in {{\mathrm{Id}}}{\mathbf {R}}$. If $b\in J\cap J^*$ then $b^2=0$ whence $b=0$ which shows $J\cap J^*=\{0\}$. Conversely, if $J\cap K=\{0\}$ then $jk\in J\cap K=\{0\}$ for all $j\in J$ and $k\in K$ and hence $K\subseteq J^*$. This shows that $J^*$ is the pseudocomplement of *J* in ${{\mathrm{\mathbf {Id}}}}{\mathbf {R}}$ completing the proof of the lemma. $\square$

### Theorem 3.7

Let $${\mathbf {R}}=(R,+,\cdot ,0)$$ be a commutative semiring. Then $${{\mathrm{\mathbf {Id}}}}^*{\mathbf {R}}$$ is an ortholattice if and only if $$I^{**}=I$$ for all $$I\in {{\mathrm{Id}}}{\mathbf {R}}$$ and $${\mathbf {R}}$$ has no 2-nilpotent element.

### Proof

First assume $${{\mathrm{\mathbf {Id}}}}^*{\mathbf {R}}$$ to be an ortholattice. Then $$I^{**}=I$$ for all $$I\in {{\mathrm{Id}}}{\mathbf {R}}$$. Moreover, if $$a\in R$$ and $$a^2=0$$ then $$a\in I(a)\cap I(a)^*=\{0\}$$ and hence $$a=0$$. Conversely, assume $$I^{**}=I$$ for all $$I\in {{\mathrm{Id}}}{\mathbf {R}}$$ and $${\mathbf {R}}$$ to have no 2-nilpotent element. Let $$J\in {{\mathrm{Id}}}{\mathbf {R}}$$. According to Lemma 3.6, $$J\cap J^*=\{0\}$$. Now, according to (i) of Lemma 3.3,

$$\begin{aligned} J+J^*=(J+J^*)^{**}=(J^*\cap J^{**})^*=(J^*\cap J)^*=\{0\}^*=R. \end{aligned}$$

Finally, the de Morgan laws hold because of Lemma 3.2. $\square$

The following two examples show which role the existence of 2-nilpotent elements plays for the fact of annihilators to be complements.

### Example 3.8

Let $${\mathbf {R}}=(R,+,\cdot ,0)$$ denote the commutative semiring with $$R:=\{0,a,b,c,$$$$d,e\}$$ and

$$\begin{aligned} \begin{array}{c|cccccc} + &{}\quad 0 &{}\quad a &{}\quad b &{}\quad c &{}\quad d &{}\quad e \\ \hline 0 &{}\quad 0 &{}\quad a &{}\quad b &{}\quad c &{}\quad d &{}\quad e \\ a &{}\quad a &{}\quad b &{}\quad 0 &{}\quad d &{}\quad e &{}\quad c \\ b &{}\quad b &{}\quad 0 &{}\quad a &{}\quad e &{}\quad c &{}\quad d \\ c &{}\quad c &{}\quad d &{}\quad e &{}\quad c &{}\quad d &{}\quad e \\ d &{}\quad d &{}\quad e &{}\quad c &{}\quad d &{}\quad e &{}\quad c \\ e &{}\quad e &{}\quad c &{}\quad d &{}\quad e &{}\quad c &{}\quad d \end{array} \quad \begin{array}{c|cccccc} \cdot &{}\quad 0 &{}\quad a &{}\quad b &{}\quad c &{}\quad d &{}\quad e \\ \hline 0 &{}\quad 0 &{}\quad 0 &{}\quad 0 &{}\quad 0 &{}\quad 0 &{}\quad 0 \\ a &{}\quad 0 &{}\quad a &{}\quad b &{}\quad 0 &{}\quad a &{}\quad b \\ b &{}\quad 0 &{}\quad b &{}\quad a &{}\quad 0 &{}\quad b &{}\quad a \\ c &{}\quad 0 &{}\quad 0 &{}\quad 0 &{}\quad c &{}\quad c &{}\quad c \\ d &{}\quad 0 &{}\quad a &{}\quad b &{}\quad c &{}\quad d &{}\quad e \\ e &{}\quad 0 &{}\quad b &{}\quad a &{}\quad c &{}\quad e &{}\quad d \end{array} \end{aligned}$$

**Fig. 2:**
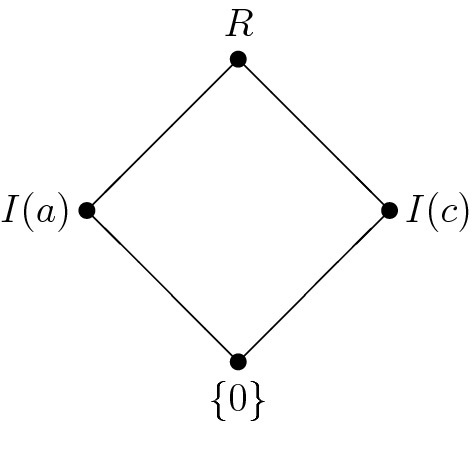
The lattice of ideals of $\mathbf R$ from Example 3.8

Then ${\mathbf {R}}$ is not a ring. Its ideals are $\{0\}$, *I*(*a*), *I*(*c*) and *R* where $I(a)=\{0,a,b\}$ and $I(c)=\{0,c\}$. It is easy to check that $\{0\}^*=R$, $I(a)^*=I(c)$, $I(c)^*=I(a)$ and $R^*=\{0\}$. Moreover, ${\mathbf {R}}$ does not contain a 2-nilpotent element. Thus, for each ideal *I* of ${\mathbf {R}}$, $I^*$ is a complement of *I* in ${{\mathrm{\mathbf {Id}}}}{\mathbf {R}}$. The Hasse diagram of ${{\mathrm{\mathbf {Id}}}}{\mathbf {R}}$ is depicted in Fig. 2.

### Example 3.9

Let $${\mathbf {R}}=(R,+,\cdot ,0)$$ denote the commutative semiring with $$R:=\{0,a,b,c,$$$$d,e,f,g\}$$,

$$\begin{aligned} \begin{array}{c|cccccccc} + &{}\quad 0 &{}\quad a &{}\quad b &{}\quad c &{}\quad d &{}\quad e &{}\quad f &{}\quad g \\ \hline 0 &{}\quad 0 &{}\quad a &{}\quad b &{}\quad c &{}\quad d &{}\quad e &{}\quad f &{}\quad g \\ a &{}\quad a &{}\quad b &{}\quad c &{}\quad 0 &{}\quad e &{}\quad f &{}\quad g &{}\quad d \\ b &{}\quad b &{}\quad c &{}\quad 0 &{}\quad a &{}\quad f &{}\quad g &{}\quad d &{}\quad e \\ c &{}\quad c &{}\quad 0 &{}\quad a &{}\quad b &{}\quad g &{}\quad d &{}\quad e &{}\quad f \\ d &{}\quad d &{}\quad e &{}\quad f &{}\quad g &{}\quad d &{}\quad e &{}\quad f &{}\quad g \\ e &{}\quad e &{}\quad f &{}\quad g &{}\quad d &{}\quad e &{}\quad f &{}\quad g &{}\quad d \\ f &{}\quad f &{}\quad g &{}\quad d &{}\quad e &{}\quad f &{}\quad g &{}\quad d &{}\quad e \\ g &{}\quad g &{}\quad d &{}\quad e &{}\quad f &{}\quad g &{}\quad d &{}\quad e &{}\quad f \end{array} \quad \begin{array}{c|cccccccc} \cdot &{}\quad 0 &{}\quad a &{}\quad b &{}\quad c &{}\quad d &{}\quad e &{}\quad f &{}\quad g \\ \hline 0 &{}\quad 0 &{}\quad 0 &{}\quad 0 &{}\quad 0 &{}\quad 0 &{}\quad 0 &{}\quad 0 &{}\quad 0 \\ a &{}\quad 0 &{}\quad a &{}\quad b &{}\quad c &{}\quad 0 &{}\quad a &{}\quad b &{}\quad c \\ b &{}\quad 0 &{}\quad b &{}\quad 0 &{}\quad b &{}\quad 0 &{}\quad b &{}\quad 0 &{}\quad b \\ c &{}\quad 0 &{}\quad c &{}\quad b &{}\quad a &{}\quad 0 &{}\quad c &{}\quad b &{}\quad a \\ d &{}\quad 0 &{}\quad 0 &{}\quad 0 &{}\quad 0 &{}\quad d &{}\quad d &{}\quad d &{}\quad d \\ e &{}\quad 0 &{}\quad a &{}\quad b &{}\quad c &{}\quad d &{}\quad e &{}\quad f &{}\quad g \\ f &{}\quad 0 &{}\quad b &{}\quad 0 &{}\quad b &{}\quad d &{}\quad f &{}\quad d &{}\quad f \\ g &{}\quad 0 &{}\quad c &{}\quad b &{}\quad a &{}\quad d &{}\quad g &{}\quad f &{}\quad e \end{array} \end{aligned}$$

**Fig. 3:**
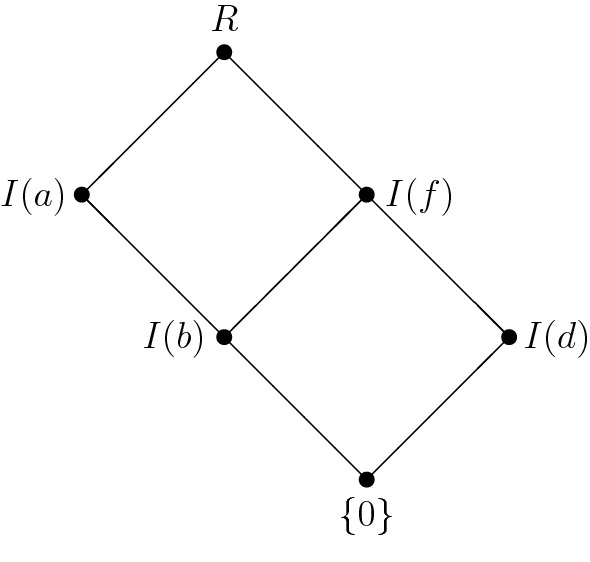
The lattice of ideals of $\mathbf R$ from Example 3.9

It is easy to check that ${\mathbf {R}}$ is not a ring. ${\mathbf {R}}$ has the following ideals: $\{0\}$, *I*(*a*), *I*(*b*), *I*(*d*), *I*(*f*) and *R* where $I(a)=\{0,a,b,c\}$, $I(b)=\{0,b\}$, $I(d)=\{0,d\}$ and $I(f)=\{0,b,d,f\}$. The Hasse diagram of ${{\mathrm{\mathbf {Id}}}}{\mathbf {R}}$ is visualized in Fig. 3.

The element *b* is 2-nilpotent and hence, according to Theorem 3.7, ${{\mathrm{\mathbf {Id}}}}^*{\mathbf {R}}$ is not an ortholattice as can be seen from $I(b)\vee I(b)^*=I(b)\vee I(f)=I(f)\ne R$.

It is easy to see that $$\{0\}^{**}\ne \{0\}$$ if and only if there exists some $$x\in R{\setminus }\{0\}$$ with $$xR=\{0\}$$. Hence, if there exists such an element *x*, then $${{\mathrm{\mathbf {Id}}}}^*{\mathbf {R}}$$ is not an ortholattice.

If ${{\mathrm{\mathbf {Ann}}}}^*{\mathbf {R}}$ is considered instead of ${{\mathrm{\mathbf {Id}}}}^*{\mathbf {R}}$, then the condition for being an ortholattice can be simplified as follows:

### Theorem 3.10

Let ${\mathbf {R}}=(R,+,\cdot ,0)$ be a commutative semiring. Then ${{\mathrm{\mathbf {Ann}}}}^*{\mathbf {R}}$ is an ortholattice if and only if there exists no $a\in R{\setminus } R^*$ with $a^2=0$.

### Proof

First assume $${{\mathrm{\mathbf {Ann}}}}^*{\mathbf {R}}$$ to be an ortholattice. If $$a\in R$$ and $$a^2=0$$ then $$a\in I(a)^*\cap I(a)^{**}=R^*$$. Conversely, assume that there exists no $$b\in R{\setminus } R^*$$ with $$b^2=0$$. Let $$J\in {{\mathrm{Ann}}}{\mathbf {R}}$$. If $$c\in J\cap J^*$$ then $$c^2=0$$ whence $$c\in R^*$$ which shows $$J\cap J^*=R^*$$. Finally, according to (i) of Lemma 3.3,

$$\begin{aligned} J\vee J^*=(J+J^*)^{**}=(J^*\cap J^{**})^*=(J^*\cap J)^*=R^{**}=R \end{aligned}$$

completing the proof of the theorem. $\square$

It is natural to ask under which condition $$^{**}$$ is a homomorphism from $${{\mathrm{\mathbf {Id}}}}^*{\mathbf {R}}$$ onto $${{\mathrm{\mathbf {Ann}}}}^*{\mathbf {R}}$$. A sufficient condition is presented by the following theorem.

### Theorem 3.11

Let ${\mathbf {R}}=(R,+,\cdot ,0)$ be a commutative semiring and assume

4 $$\begin{aligned} (I\cap J)^{**}=I^{**}\cap J^{**}\text { for all }I,J\in {{\mathrm{Id}}}{\mathbf {R}}. \end{aligned}$$

Then $$^{**}$$ is a homomorphism from $${{\mathrm{\mathbf {Id}}}}^*{\mathbf {R}}$$ onto $${{\mathrm{\mathbf {Ann}}}}^*{\mathbf {R}}$$. Moreover, we have

$$\begin{aligned} \left( \sum _{s\in S}I_s\right) ^{**}&=\bigvee I_s^{**}\text { and} \\ \left( \bigcap _{s\in S}I_s\right) ^{**}&\subseteq \bigcap _{s\in S}I_s^{**} \end{aligned}$$

for every family $(I_s;s\in S)$ of ideals of ${\mathbf {R}}$.

### Proof

Let $$I,J\in {{\mathrm{Id}}}{\mathbf {R}}$$. Then

$$\begin{aligned} (I+J)^{**}&=(I^*\cap J^*)^*=(I^{***}\cap J^{ ***})^*\\&=(I^{**}+J^{**})^{**}=I^{**}\vee J^{**}, \\ (I\cap J)^{**}&=I^{**}\cap J^{**}, \\ (I^*)^{**}&=(I^{**})^*, \\ \{0\}^{**}&=R^*\text { and} \\ R^{**}&=R. \end{aligned}$$

Moreover, we have

$$\begin{aligned} \left( \sum _{s\in S}I_s\right) ^{**}= & {} \left( \bigcap _{s\in S}I_s^*\right) ^*=\left( \bigcap I_s^{***}\right) ^*\\&=\left( \sum _{s\in S}I_s^{**}\right) ^{**}=\bigvee _{s\in S}I_s^{**}. \end{aligned}$$

for every family $(I_s;s\in S)$ of ideals of ${\mathbf {R}}$. The rest of the proof is clear. $\square$

For any commutative semiring ${\mathbf {R}}$ and any $\Theta \in {{\mathrm{Con}}}{\mathbf {R}}$ we call $[0]\Theta$ the *kernel* of $\Theta$.

In contrast to the case of commutative rings, an ideal of a commutative semiring ${\mathbf {R}}$ need not be the kernel of some congruence on ${\mathbf {R}}$. We have only $[0]\Theta \in {{\mathrm{Id}}}{\mathbf {R}}$ provided $\Theta \in {{\mathrm{Con}}}{\mathbf {R}}$. A weaker result holds for the so-called *Bourne congruence* induced by an ideal *I* of ${\mathbf {R}}$:

### Theorem 3.12

Let *I* be some ideal of a commutative semiring ${\mathbf {R}}=(R,+,\cdot ,0)$ and put

$$\begin{aligned} \Theta (I):=\{(x,y)\in R^2\mid \text {there exist }a,b\in I\text { with }x+a=y+b\}. \end{aligned}$$

The $$\Theta (I)\in {{\mathrm{Con}}}{\mathbf {R}}$$ and $$I\subseteq [0]\Theta (I)$$.

The proof is easy (cf. Proposition 6.50 in Golan 1999) and therefore omitted.

## Ideals and congruence kernels in Łukasiewicz semirings

In the following we investigate commutative semirings with a unary operation $'$. For such extended semirings we study complements in the lattice of ideals which are not derived by annihilators as above, but by means of this unary operation.

The concept of a *Łukasiewicz semiring* was introduced in Bonzio et al. (2016) and Chajda et al. (2018) as an algebraic semantic of a certain non-classical logic used in quantum mechanics. The motivation can be found in Chajda et al. (2018) and Bonzio et al. (2016).

Namely, within the logic of quantum mechanics effects are described by means of the so-called effect algebras. Z. Riečanová proved that every lattice effect algebra is built up by blocks which are in fact MV-algebras. MV-algebras were introduced in the 1950’s by C. C. Chang as an algebraic axiomatization of Łukasiewicz many-valued logics. As shown in Chajda et al. (2018), MV-algebras are in one-to-one correspondence with Łukasiewicz semirings. Using these semirings instead of MV-algebras, one can apply the theory of semirings developed, e.g. in Golan (1999), i.e. the theory of ideals and annihilators in particular.

A *Łukasiewicz semiring* is an algebra $\mathbf R=(R,+,\cdot ,{}',0,1)$ of type (2, 2, 1, 0, 0) such that

(i) $(R,+,\cdot ,0,1)$ is a unitary and idempotent commutative semiring,
(ii) $$x+1\approx 1$$,
(iii) $$(x'y)'y\approx (y'x)'x$$,
(iv) $'$ is an antitone involution of the poset $(R,\le )$ where $\le$ denotes the partial order relation corresponding to the bounded join-semilattice $(R,+,0,1)$.

The special case $y=0$ in (iii) yields $xx'\approx 0$. As shown in Chajda et al. (2018), the following identity holds in every Łukasiewicz semiring:

$$\begin{aligned} x+y\approx ((xy')'y')'. \end{aligned}$$

If $a\in R$ satisfies $a^2=a$, then the previous identity yields

$$\begin{aligned} a+a'=((aa)'a)'=(a'a)'=0'=1. \end{aligned}$$

Since Łukasiewicz semirings are commutative semirings, we use the same definition of an ideal as before.

### Lemma 4.1

Let ${\mathbf {R}}=(R,+,\cdot ,{}',0,1)$ be a Łukasiewicz semiring and *I* a subset of *R* with $0\in I$ satisfying

5 $$\begin{aligned} \text {If }x\in R\text { and }y,xy'\in I\text {, then }x\in I. \end{aligned}$$

Then $$I\in {{\mathrm{Id}}}{\mathbf {R}}$$.

### Proof

Let $a,b\in I$ and $c\in R$. We have $(a'b)b'=a'bb'=0\in I$ whence $a'b\in I$ according to (5). Moreover, $(a+b)a'=aa'+ba'=a'b\in I$ whence $a+b\in I$ again according to (5). Finally, $(ac)a'=aa'c=0\in I$ whence $ac\in I$ according to (5) completing the proof of the lemma. $\square$

For every Łukasiewicz semiring ${\mathbf {R}}=(R,+,\cdot ,{}',0,1)$ let ${{\mathrm{CK}}}({\mathbf {R}})$ denote the set of all subsets *I* of *R* with $0\in I$ satisfying (5) and the following condition:

$$\begin{aligned} x,y,z\in R\text { and }xy'\in I\text { imply }xz(yz)'\in I. \end{aligned}$$

According to Lemma 4.1, ${{\mathrm{CK}}}({\mathbf {R}})\subseteq {{\mathrm{Id}}}\mathbf R$. Put ${{\mathrm{\mathbf {CK}}}}{\mathbf {R}}:=({{\mathrm{CK}}}{\mathbf {R}},\subseteq )$.

### Lemma 4.2

Let ${\mathbf {R}}=(R,+,\cdot ,{}',0,1)$ be a Łukasiewicz semiring and $\Theta \in {{\mathrm{Con}}}{\mathbf {R}}$. Then $[0]\Theta \in {{\mathrm{CK}}}{\mathbf {R}}$.

### Proof

Let $a,b,c\in R$. Obviously, $0\in [0]\Theta$. If $b,ab'\in [0]\Theta$ then $a=a1=a0'\in [ab']\Theta =[0]\Theta$. Finally, if $ab'\in [0]\Theta$ then

$$\begin{aligned} ac(bc)'= & {} 1ac(bc)'=0'ac(bc)'\in [(b'a)'ac(bc)']\Theta \\= & {} [(a'b)'bc(bc)']\Theta =[0]\Theta . \end{aligned}$$

$$\square $$

Next we prove that ${{\mathrm{CK}}}{\mathbf {R}}$ is just the set of congruence kernels of ${\mathbf {R}}$.

### Theorem 4.3

Let ${\mathbf {R}}=(R,+,\cdot ,{}',0,1)$ be a Łukasiewicz semiring and $I\in {{\mathrm{CK}}}{\mathbf {R}}$. Put

$$\begin{aligned} \Theta (I):=\{(x,y)\in R^2\mid xy',x'y\in I\}. \end{aligned}$$

Then $$\Theta (I)\in {{\mathrm{Con}}}{\mathbf {R}}$$.

### Proof

Let $a,b,c\in R$. It is evident that $\Theta (I)$ is reflexive and symmetric. Now assume $(a,b),(b,c)\in \Theta (I)$, i.e. $ab',a'b,bc',b'c\in I$. Since

$$\begin{aligned} ac'(bc')'=a(bc')'c'=a(cb')'b'\in I \end{aligned}$$

and

$$\begin{aligned} a'c(b'c)'=a'(b'c)'c=a'(c'b)'b\in I \end{aligned}$$

we have $ac',a'c\in I$ according to (5), i.e. $(a,c)\in \Theta (I)$ showing transitivity of $\Theta (I)$. Next assume $(a,b)\in \Theta (I)$, i.e. $ab',a'b\in I$. Then

$$\begin{aligned} ac(bc)'=a(bc)'c=a(c'b')'b'\in I \end{aligned}$$

and

$$\begin{aligned} (ac)'bc=(ac)'cb=(c'a')'a'b\in I \end{aligned}$$

showing $(ac,bc)\in \Theta (I)$ according to (5). Obviously, $\Theta (I)$ is compatible with $'$. Finally, $(a,b)\in \Theta (I)$ implies $(a+c,b+c)=(((ac')'c')',((bc')'c')')\in \Theta (I)$ completing the proof of the lemma. $\square$

### Theorem 4.4

Let ${\mathbf {R}}=(R,+,\cdot ,{}',0,1)$ be a Łukasiewicz semiring. Then ${{\mathrm{\mathbf {CK}}}}{\mathbf {R}}$ is isomorphic to ${{\mathrm{\mathbf {Con}}}}{\mathbf {R}}:=({{\mathrm{Con}}}\mathbf R,\subseteq )$ and hence a complete lattice. The infimum in ${{\mathrm{\mathbf {CK}}}}{\mathbf {R}}$ coincides with the set-theoretical intersection. Moreover, the correspondence described in Lemma 4.2 and Theorem 4.3 is one-to-one.

### Proof

Let $a,b\in R$, $\Phi ,\Psi \in {{\mathrm{Con}}}{\mathbf {R}}$ and $I,J\in {{\mathrm{CK}}}\mathbf R$. If $(a,b)\in \Theta ([0]\Phi )$ then $ab',a'b\in [0]\Phi$ and hence

$$\begin{aligned} a=1a=0'a\,\Phi \,(b'a)'a=(a'b)'b\,\Phi \,0'b=1b=b. \end{aligned}$$

If, conversely, $(a,b)\in \Phi$ then $ab',a'b\in [aa']\Phi =[0]\Phi$ and hence $(a,b)\in \Theta ([0]\Phi )$. This shows $\Theta ([0]\Phi )=\Phi$. Moreover, the following are equivalent: $a\in [0]\Theta (I)$; $a0^{\prime },a^{\prime }0\in I$; $a\in I$. This shows $[0]\Theta (I)=I$. Finally, $\Phi \subseteq \Psi$ implies $[0]\Phi \subseteq [0]\Psi$, and $I\subseteq J$ implies $\Theta (I)\subseteq \Theta (J)$. That the infimum in ${{\mathrm{\mathbf {CK}}}}{\mathbf {R}}$ coincides with set-theoretical intersection follows from $\bigcap \nolimits _{i\in I}([0]\Theta _i)=[0](\bigcap \nolimits _{i\in I}\Theta _i)$ for every family $\Theta _i,i\in I,$ of congruences on ${\mathbf {R}}$. $\square$

Since in a Łukasiewicz semiring ${\mathbf {R}}=(R,+,\cdot ,{}',0,1)$ we have $x\vee y=x+y$ and since $'$ is an antitone involution on the poset $(R,\le )$ corresponding to the join-semilattice $(R,+)$, we can use De Morgan laws and obtain $x\wedge y=(x'+y')'$.

The following lemma lists some important properties of Łukasiewicz semirings.

### Lemma 4.5

Let ${\mathbf {R}}=(R,+,\cdot ,{}',0,1)$ be a Łukasiewicz semiring and $a,b,c\in R$. Then the following hold:

(i) If $$a\le b$$ then $$ac\le bc$$,
(ii) $a\le b$ if and only if $ab'=0$,
(iii) if $a+b=1$ and $ab=0$ then $b=a'$,
(iv) $$ab\le a\wedge b$$,
(v) $$I(a)=aR$$.

### Proof

(i) If $a\le b$ then $a+b=b$ and hence $ac+bc=(a+b)c=bc$ whence $ac\le bc$.
(ii) If $a\le b$ then $ab'\le bb'=0$ according to (i) and hence $ab'=0$. Conversely, if $ab'=0$ then
  $$\begin{aligned} a+b=((ab')'b')'=(0'b')'=(1b')'=(b')'=b, \end{aligned}$$
  i.e. $$a\le b$$.
(iii) If $a+b=1$ and $ab=0$ then $b\le a'$ according to (ii) and
  $$\begin{aligned} a'b'=1a'b'=(a+b)a'b'=aa'b+abb'=0+0=0 \end{aligned}$$
  whence $b\ge a'$ again according to (ii) and hence $b=a'$.
(iv) We have
  $$\begin{aligned} ab=(a')'b=(a'1)'b\le (a'b)'b=(a'+b')'=a\wedge b \end{aligned}$$
  according to (i).
(v) This follows since ${\mathbf {R}}$ is unitary.

$$\square $$

For any two ideals *I*, *J* of a commutative semiring $\mathbf R=(R,+,\cdot ,0)$ we have $IJ\subseteq I\cap J$, but in general we do not have equality. However, in some cases equality follows.

### Lemma 4.6

Let ${\mathbf {R}}=(R,+,\cdot ,0,1)$ be a unitary commutative semiring and $I,J\in {{\mathrm{Id}}}{\mathbf {R}}$ satisfying $I+J=R$ and $IJ=\{0\}$. Then $IJ=I\cap J$.

### Proof

Since $I+J=R$ there exists $a\in I$ and $b\in J$ with $a+b=1$. Now $x=x1=x(a+b)=xa+xb=0$ for all $x\in I\cap J$ completing the proof of the lemma. $\square$

### Theorem 4.7

Let ${\mathbf {R}}=(R,+,\cdot ,{}',0,1)$ be a Łukasiewicz semiring and $I,J\in {{\mathrm{Id}}}{\mathbf {R}}$. Then *I* and *J* are complements of each other in ${{\mathrm{\mathbf {Id}}}}{\mathbf {R}}$ if and only if there exists some $a\in R$ with $a+a'=1$, $I(a)=I$ and $I(a')=J$.

### Proof

First assume *I* and *J* to be complements of each other in ${{\mathrm{\mathbf {Id}}}}{\mathbf {R}}$. Since $I+J=R$ there exist $a\in I$ and $b\in J$ with $a+b=1$. Since $IJ\subseteq I\cap J=\{0\}$ we have $ab=0$. According to (iii) of Lemma 4.5 we conclude $b=a'$. If $c\in I$ then $ca'\in IJ=\{0\}$, i.e. $ca'=0$, and hence $c=c1=c(a+a')=ca+ca'=ca+0=ca\in I(a)$ proving $I\subseteq I(a)$. Since the converse inclusion is evident, we have $I(a)=I$. If $d\in J$ then $ad\in IJ=\{0\}$, i.e. $ad=0$, and hence $d=1d=(a+a')d=ad+a'd=0+a'd=a'd\in I(a')$ proving $J\subseteq I(a')$. Since the converse inclusion is evident, we have $I(a')=J$. Conversely, assume $e\in R$, $e+e'=1$, $I(e)=I$ and $I(e')=J$. Since $e+e'=1$, we have $x=x1=x(e+e')=xe+xe'\in I+J$ for all $x\in R$, i.e. $I+J=R$. Now $IJ=ReRe'=\{0\}$. According to Lemma 4.6, $I\cap J=\{0\}$ which shows that *I* and *J* are complements of each other in ${{\mathrm{\mathbf {Id}}}}{\mathbf {R}}$. $\square$

## Direct product of a commutative semiring and a finite unitary Boolean ring

If ${\mathbf {R}}_1=(R_1,+,\cdot ,0)$ and ${\mathbf {R}}_2=(R_2,+,\cdot ,0)$ are commutative semirings, $I_1\in {{\mathrm{Id}}}{\mathbf {R}}_1$ and $I_2\in {{\mathrm{Id}}}{\mathbf {R}}_2$ then $I_1\times I_2\in {{\mathrm{Id}}}(\mathbf R_1\times {\mathbf {R}}_2)$. An *ideal* of ${\mathbf {R}}_1\times \mathbf R_2$ which is not of this form is called *skew*.

If ${\mathbf {R}}_1\times {\mathbf {R}}_2$ has no skew ideals, then ${{\mathrm{\mathbf {Id}}}}({\mathbf {R}}_1\times {\mathbf {R}}_2)\cong {{\mathrm{\mathbf {Id}}}}\mathbf R_1\times {{\mathrm{\mathbf {Id}}}}{\mathbf {R}}_2$ and hence ${{\mathrm{\mathbf {Id}}}}({\mathbf {R}}_1\times \mathbf R_2)$ is complemented if and only if ${{\mathrm{\mathbf {Id}}}}{\mathbf {R}}_1$ and ${{\mathrm{\mathbf {Id}}}}{\mathbf {R}}_2$ have this property. We are going to show that if one of two given commutative semirings is a finite unitary Boolean ring then the lattice of ideals of their direct product turns out to be directly decomposable. Recall that a *ring* is called *Boolean* if it satisfies the identity $x^2\approx x$. It is well known that such rings are commutative and satisfy the identity $x+x\approx 0$.

### Lemma 5.1

Let ${\mathbf {R}}=(R,+,\cdot ,0)$ be a commutative semiring and $\mathbf B=(B,+,\cdot ,0)$ a finite unitary Boolean ring. Then $\mathbf R\times {\mathbf {B}}$ has no skew ideals.

### Proof

If $|B|=1$ then the assertion of the lemma is trivial. Now assume $|B|=2$. Then ${\mathbf {B}}\cong {\mathbb {Z}}_2$. Let $I\in {{\mathrm{Id}}}(\mathbf R\times {\mathbb {Z}}_2)$. If $I\subseteq R\times \{0\}$ then *I* is not a skew ideal. Hence, assume $I\not \subseteq R\times \{0\}$. Then there exists some $a\in R$ with $(a,1)\in I$. We conclude $(0,1)=(a,1)(0,1)\in I$. Let $b\in R$. If $(b,0)\in I$ then $(b,1)=(b,0)+(0,1)\in I$. If, conversely, $(b,1)\in I$ then $(b,0)=(b,1)+(0,1)\in I$. This shows that $(b,0)\in I$ if and only if $(b,1)\in I$. Hence, *I* is not a skew ideal of $\mathbf R\times {\mathbb {Z}}_2$. If, finally, $|B|>2$ then ${\mathbf {B}}$ is a direct product of $n\ge 2$ copies of ${\mathbb {Z}}_2$ and the assertion follows by induction on *n*. $\square$

**Fig. 4:**
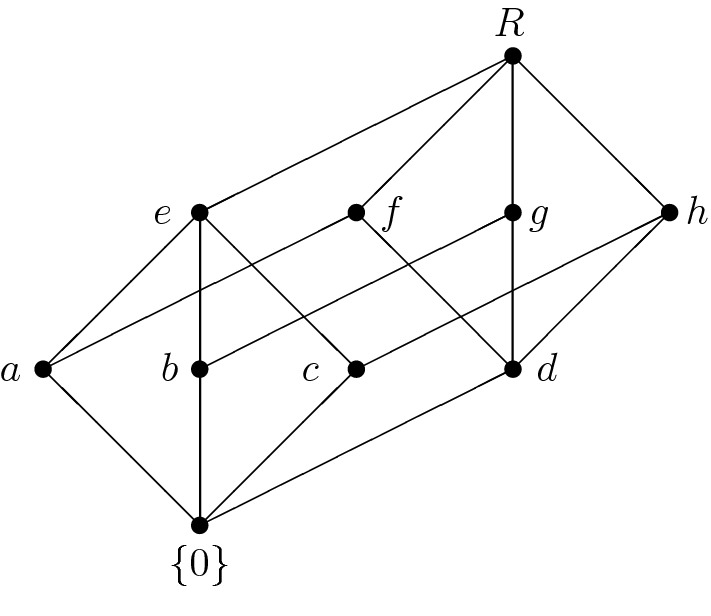
The lattice of ideals of $\mathbf R$ from Example 5.3

**Fig. 5:**
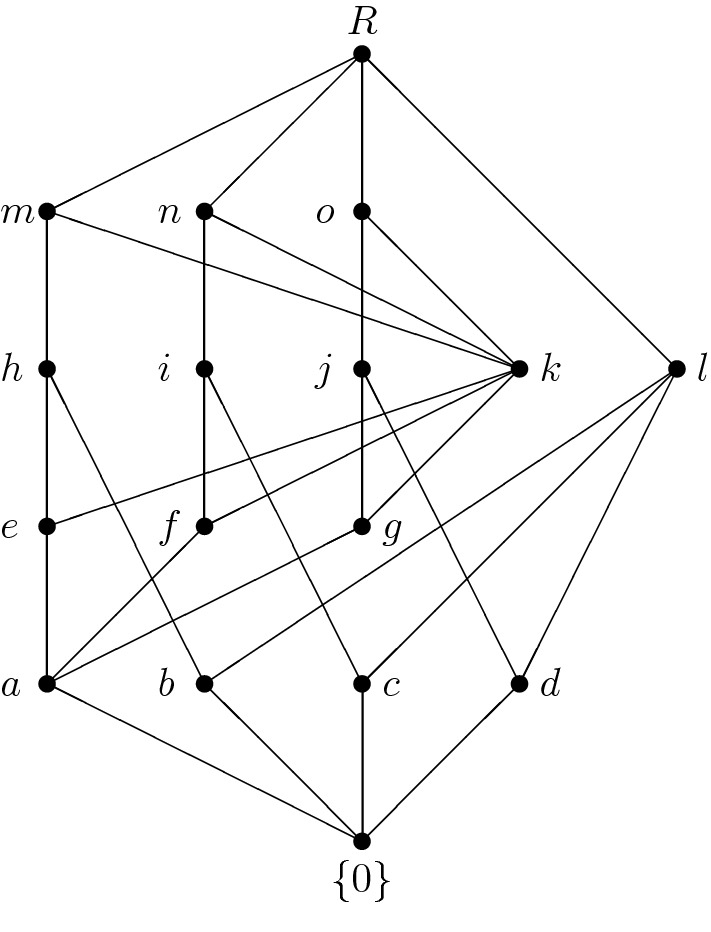
The lattice of ideals of $\mathbf R_1$ from Example 5.3

### Corollary 5.2

If ${\mathbf {R}}$ is a commutative semiring whose lattice of ideals is complemented or modular or distributive and ${\mathbf {B}}$ is a finite unitary Boolean ring, then ${\mathbf {R}}\times {\mathbf {B}}$ is a commutative semiring whose lattice of ideals is complemented or modular or distributive, respectively.

Hence, if ${\mathbf {R}}$ denotes the commutative semiring from Example 2.2 whose lattice of ideals is complemented (but complements do not coincide with annihilators in general) and ${\mathbf {B}}$ is a finite unitary Boolean ring, then $\mathbf R\times {\mathbf {B}}$ is a commutative semiring whose lattice of ideals is complemented, too. Hence, using our procedure, we may produce infinitely many commutative semirings having the mentioned property.

We are going to show that the finite unitary Boolean ring in the previous corollary cannot be substituted by a bounded distributive lattice even in the case when this finite unitary Boolean ring is the two-element one.

### Example 5.3

Consider the Kleinian four-element group as the additive group of a zero-ring. Denote this ring by ${\mathbf {K}}$. Now consider the two-element unitary Boolean ring ${\mathbf {B}}$ and put $\mathbf R:={\mathbf {K}}\times {\mathbf {B}}$. Since ${\mathbf {R}}$ is a ring, its lattice of ideals is modular (even complemented) and its Hasse diagram is shown in Fig. 4.

The structure of ${{\mathrm{\mathbf {Id}}}}{\mathbf {R}}$ follows directly from Lemma 5.1 since

$$\begin{aligned} {{\mathrm{\mathbf {Id}}}}{\mathbf {R}}={{\mathrm{\mathbf {Id}}}}({\mathbf {K}}\times {\mathbf {B}}_2)\cong {{\mathrm{\mathbf {Id}}}}\mathbf K\times {{\mathrm{\mathbf {Id}}}}{\mathbf {B}}_2\cong {\mathbf {M}}_3\times {\mathbf {2}}. \end{aligned}$$

If, however, we substitute ${\mathbf {B}}$ by the two-element distributive lattice (considered as a commutative semiring), we obtain a semiring ${\mathbf {R}}_1$ which is not a ring and whose lattice of ideals contains skew ideals. This lattice of ideals is not modular. Its Hasse diagram is depicted in Fig. 5.

However, ${{\mathrm{\mathbf {Id}}}}{\mathbf {R}}_1$ is complemented:

$\{0\}$ is a complement of *R*,

*b* is a complement of *i*, *j*, *n* and *o*,

*c* is a complement of *h*,

*d* is a complement of *m*, and

*l* is a complement of *a*, *e*, *f*, *g* and *k*.
